# Dissecting how psychopathic traits are linked to learning in different contexts: A multilevel computational and electrophysiological approach

**DOI:** 10.3758/s13415-025-01295-z

**Published:** 2025-07-23

**Authors:** Josi M. A. Driessen, Andreea O. Diaconescu, Dimana V. Atanassova, Jan K. Buitelaar, Roy P. C. Kessels, Jeffrey C. Glennon, Inti A. Brazil

**Affiliations:** 1 Donders Institute for Brain, Cognition and Behaviour, Radboud University, Nijmegen, The Netherlands; 2 Krembil Centre for Neuroinformatics at CAMH, University of Toronto, Toronto, Canada; 3 Conway Institute of Biomedical and Biomolecular Research, School of Medicine, University College Dublin, Dublin, Ireland; 4 Forensic Psychiatric Centre Pompestichting, Nijmegen, The Netherlands; 5 Department of Cognitive Neuroscience, Donders Institute for Brain, Cognition, and Behaviour, Radboudumc, Nijmegen, The Netherlands

**Keywords:** Psychopathy, Associative learning, Social learning, Computational modelling, Theta power

## Abstract

Previous studies suggest that elevated psychopathic traits, linked to social norm violations and personal gain-seeking, may be caused by impairments in associative learning. Recent advances in computational modelling offer insight into the unobservable processes that are thought to underly associative learning. Using such a model, the present study investigated the associations between psychopathic traits in a nonoffender sample and the cognitive computations underlying adaptive behavior during associative learning. We also investigated the potential engagement of adaptive control processes by measuring oscillatory theta activity in the prefrontal cortex. Participants performed a reinforcement learning task in which the trade-off between using social and nonsocial information affected task performance and the associated monetary reward. The findings indicated that increasing levels of psychopathic traits co-occurred with reduced learning from social information and suggested that antisocial traits were linked to a reduced ability to track changes in the trustworthiness of social advice over time. This did not affect the preference for one information source and the risk taken to obtain a high reward. Furthermore, midfrontal theta power was negatively linked to levels of psychopathic traits, aligning with indications that theta is involved in volatility tracking of social information. Importantly, we consider that the task design may reflect reduced sensitivity to secondary, rather than specifically social information. The current study provides support for a relationship between associative learning, theta power, and psychopathic traits and contributes to our understanding of the mechanisms that may explain reduced responsiveness to current treatment interventions in individuals with psychopathy.

**Supplementary Information:**

The online version contains supplementary material available at 10.3758/s13415-025-01295-z.

## Introduction

Psychopathy is a personality construct that entails disturbances in both interpersonal (e.g., showing superficial charm, manipulative behavior, and deceitfulness) and affective domains (e.g., being callous, lack of empathy, and guilt). These traits co-occur with the tendency to lead an erratic lifestyle (e.g., impulsivity, leading a parasitic lifestyle, irresponsibility) and to engage in antisocial behaviors (e.g., aggressiveness, juvenile delinquency, and criminal versatility) (Hare, 2003). This combination of interpersonal and affective facets is argued to be unique to psychopathy, while the lifestyle and antisocial components represent more general traits that can be found across antisocial populations (Blackburn, 2007; Neumann et al., 2007). Support for this model has been provided by several studies across a wide variety of samples (Hill et al., 2004; Kosson et al., 2002; Neumann et al., 2007). It has been demonstrated that the construct of psychopathy is dimensional in nature and consists of a constellation of traits that vary along a continuum (Gao & Raine, 2010; Hare & Neumann, 2005, 2008). This line of work has shown that psychopathic traits can be measured in both offenders and among the general community (Brandt et al., 1997; DeMatteo et al., 2006; Malterer et al., 2010; Neumann & Hare, 2008).

In clinical settings, individuals with elevated levels of psychopathy are known for their reduced ability to adequately monitor and adapt their behavior in response to treatment (Ly et al., 2016). Research has shown that these maladaptive tendencies are linked to impairments in associative learning (Blair et al., 2004; Mitchell et al., 2006; Newman & Kosson, 1986; Patterson & Newman, 1993). For instance, psychopathic offenders show deficits in learning the associations between stimuli and reinforcers that occur in temporal proximity to the stimuli (i.e., contingency learning) (Brazil et al., 2013a, b; Gregory et al., 2015; Von Borries et al., 2010). Associative learning also plays a key role in the acquisition of desirable social behaviors (Behrens et al., 2009) and seems to be one of the mechanisms through which we learn about social expectations and moral reasoning (Blair, 2013; Blair & Cipolotti, 2000). The tendency to consistently violate social norms and to behave antisocially in individuals with high levels of psychopathy points towards disturbances in social learning (Brazil et al., 2011). Indeed, individuals with psychopathic tendencies have been found to show impaired learning from social information (Blair, 2007; Brazil et al., 2011, 2013a) and fail to benefit optimally from standard socialization techniques (Wootton et al., 1997). As a consequence, they are more likely to learn to apply antisocial (moral) strategies to achieve their goals (Driessen et al., 2021b), which has a detrimental impact on their social environment and acceptance by their peer group. Importantly, significantly advancing our understanding of the underlying biopsychological mechanisms in psychopathy requires multidisciplinary research that is embedded within the most recent frameworks of associative learning.

General neuroscientific research has provided insight into the computational mechanisms that are thought to underlie associative learning (Brazil et al., 2013a, b; Behrens et al., 2008; Cohen, 2008; Oba et al., 2019; Sutton & Barto, 2018). Optimal learning requires the accurate generation of estimates (or representations) of the characteristics of each stimulus-outcome contingency (Brazil et al., 2017). Because we live in a complex and dynamic environment, these contingencies typically change over time (i.e., are volatile) (Dayan et al., 2000). Consequently, we need to continuously monitor the relationship between events and their outcomes and update our representations of such contingencies after each observation to keep the representations accurate. However, these representations will never reach perfection, because there will always be inaccuracy (or uncertainty) in how we process incoming sensory information (Findling et al., 2019). The level of volatility in the environment determines the rate at which a person learns. In stable and predictable environments, where contingencies remain consistent (i.e., low uncertainty), fewer updates are required, resulting in low learning rates. In contrast, in volatile environments, characterized by rapid changes (i.e., high uncertainty), quicker adaptations are required to update contingency estimates, resulting in higher learning rates (Behrens et al., 2007; Courville et al., 2006; Dayan et al., 2000). Furthermore, the rate at which contingency changes are perceived to occur affects our belief about the overall likelihood that the contingencies will change, indicating that these two aspects of change are hierarchically coupled (Brazil et al., 2017; Mathys et al., 2011, 2014). Thus, appropriate adaptation of behavior depends in part on our ability to learn about the rate and overall likelihood with which contingencies can change in our environment (Brazil et al., 2017) and also on how well these two processes interact (Diaconescu et al., 2020).

Importantly, behavioral adaptation can be triggered by changes in social and nonsocial factors. Previous studies have demonstrated that learning from social and nonsocial information sources is subserved by similar mechanisms, even though these are implemented in distinct neural regions (Behrens et al., 2008; King-Casas et al., 2005). Coding of information related to the volatility of such information has been linked to substructures within the anterior cingulate cortex (ACC), and the information is then combined in the medial prefrontal cortex (mPFC) to guide further adaptation of behavior (Behrens et al., 2008). Electrophysiological findings have suggested that midfrontal activation in the theta frequency band, recorded from electrodes over the mPFC, reflects a common mechanism for implementing adaptive control in situations involving uncertainty about actions and outcomes (Cavanagh et al., 2010, 2012a). Adaptive control refers to the process of monitoring ongoing actions and performance outcomes, and subsequent adjustments of behavior and learning (Ridderinkhof et al., 2004). Theta activity was found to be central to adaptive control, as it is associated with representations concerning the occurrence of reward and the learning rate (Cavanagh et al., 2010, 2012a, b; Cohen et al., 2007; Ferdinand et al., 2012; Marco-Pallares et al., 2008; Mas-Herrero & Marco-Pallarés, 2014; Talmi et al., 2013). For instance, Mas-Herrero & Marco-Pallarés demonstrated that theta band activation is linked to inaccuracies in expectations of reward (i.e., reward prediction errors) in both the acquisition of contingencies and after reversal of these contingencies. Furthermore, they found that fluctuations in theta band activity covaried with the learning rate across participants during both types of learning. Theta activation was higher during reversal learning compared with acquisition learning, which is in line with the suggestion that the mPFC, including the ACC, tracks the environmental volatility (Behrens et al., 2007). The authors proposed that increases in frontal theta activity could be analogous to the increase of mPFC activity associated with the learning rate that were demonstrated in previous fMRI studies (Behrens et al., 2007; Jocham et al., 2009; Krugel et al., 2009; Walton et al., 2004; Yoshida & Ishii, 2006). Thus, midfrontal theta can be seen as an electrophysiological marker reflecting the engagement of adaptive control processes during associative learning.

Although research on electrophysiological dynamics in the time–frequency domain in relation to psychopathy is scarce (Clark et al., 2019), some studies have highlighted a role for theta activity in information processing in psychopathic offenders. Two studies demonstrated reduced theta activity in response to affective stimuli (Eisenbarth et al., 2013; Tillem et al., 2016), which was suggested to underlie impairments in the processing and integration of sensory information (Tillem et al., 2016). Other work has examined theta band activity in relation to external correlates of psychopathy, such as externalizing behavior (Bernat et al., 2011) and antisocial behavior (Mednick et al., 1981), but did not provide support for a link between theta activity and psychopathy-related behavior. To date, there is no clear consensus on the role of theta band oscillations in psychopathy. Moreover, there are no studies that examined adaptive control processes and theta activity in relation to psychopathic traits in nonoffenders.

By examining associative learning in a group of community-dwelling individuals with varying levels of psychopathic traits with the use of computational models, we could obtain a deeper insight into the latent processes underlying associative learning that are altered as a function of distinct levels of psychopathy. The increased precision offered by such an approach can be further enhanced by additionally collecting electrophysiological measurements of the mPFC that can be used as direct measures of adaptive control during associative learning. Therefore, the present study combined computational modelling with electrophysiological measurements to investigate how levels of psychopathic traits covary with 1) the use of social and nonsocial information during associative learning, 2) the updating of representations concerning the volatility of social and nonsocial information, and 3) oscillatory theta band activation during associative learning. Based on findings pointing towards impaired associative social learning in psychopathy (Blair, 2013; Brazil et al., 2011, 2013a), we expected interpersonal-affective traits to be negatively related to the use of social advice during learning. In contrast, we predicted that lifestyle-antisocial traits should be positively linked to the use of nonsocial information during learning, based on previous studies linking high levels of impulsive-antisocial traits to hypersensitivity to reward (Buckholtz et al., 2010). At the computational level, we hypothesized that increasing levels of interpersonal-affective and antisocial traits should be linked to disturbances in the representation of volatility, based on findings suggesting that these psychopathic features are linked to an excessively large amount of inaccuracy in the representation of volatility during threat conditioning (Brazil et al., 2017). Finally, we expected reduced theta activity in relation to psychopathic traits, based on the notion that higher psychopathy levels should co-occur with diminished adaptive control during associative learning and on previous studies reporting a negative link between theta band activation and psychopathy. We refrained from generating hypotheses for individual facet scores given that these have not been explored before in relation to theta activity.

## Methods

### Participants

Based on a priori samples size calculations (power = 0.85, effect size = 0.30), 86 participants were included in this study (36 males). Participants were between 18 and 35 years of age (*M* = 24.1, *SD* = 2.8) and were native Dutch speakers. Exclusion criteria for the current study were self-reported epilepsy, head surgery, claustrophobia, history of other neurological conditions or psychiatric disease, use of psychoactive medication or substances, and pregnancy. Participants were selected from a large database (*N* = 1519, 309 men) based on their level of psychopathic traits, which was assessed with the short-form version of the self-report psychopathy checklist (SRP-SF; Dutch version) (Gordts et al., 2017). The SRP-SF is a self-report questionnaire including 29 items that need to be rated on a 5-point Likert scale. Subscale scores (interpersonal, affective, lifestyle, and antisocial) can be derived as well as a total score. A substantial amount of literature supports the validity and reliability of the SRP-SF in community samples (Gordts et al., 2017; Lilienfeld et al., 2006; Neumann et al., 2007; Neumann & Pardini, 2014). Internal consistency in the sample was high (all Cronbach’s alpha > 0.75). Participants in the large database were recruited via local advertisement and an online research participation system (SONA systems) of the Radboud University in Nijmegen. The total scores in the large sample (*N* = 1519, 309 males) were divided in quartiles to make sure that 25% of the participants ended up in the top and bottom quartiles, whereas 50% of the participants ended up in the two middle quartiles. The experimental sample consisted of 86 participants (36 males). The top and bottom quartiles were oversampled to enhance the presence of extreme scores on both sides of the distribution (8, 60), which would otherwise remain underrepresented given the normal distribution of psychopathy scores. Consequently, 20 participants (23.3%) belonged to the lowest quartile, 40 participants (46.5%) belonged to the two middle segments, and 26 participants (30.2%) belonged to the upper quartile. Note the final sample consisted of 80 participants for analyses involved electrophysiological measures, because six participants were excluded from these analyses due to major distortions in at least one of the midfrontal electrodes. All participants provided written informed consent prior to the onset of the study and received monetary compensation at the end of the study. Procedures were approved by the Ethics Committee Social Sciences of the Radboud University Nijmegen (ECSW2017 - 0805–512) and research has been performed in accordance with the Declaration of Helsinki.

### Wager task

**Fig. 1:**
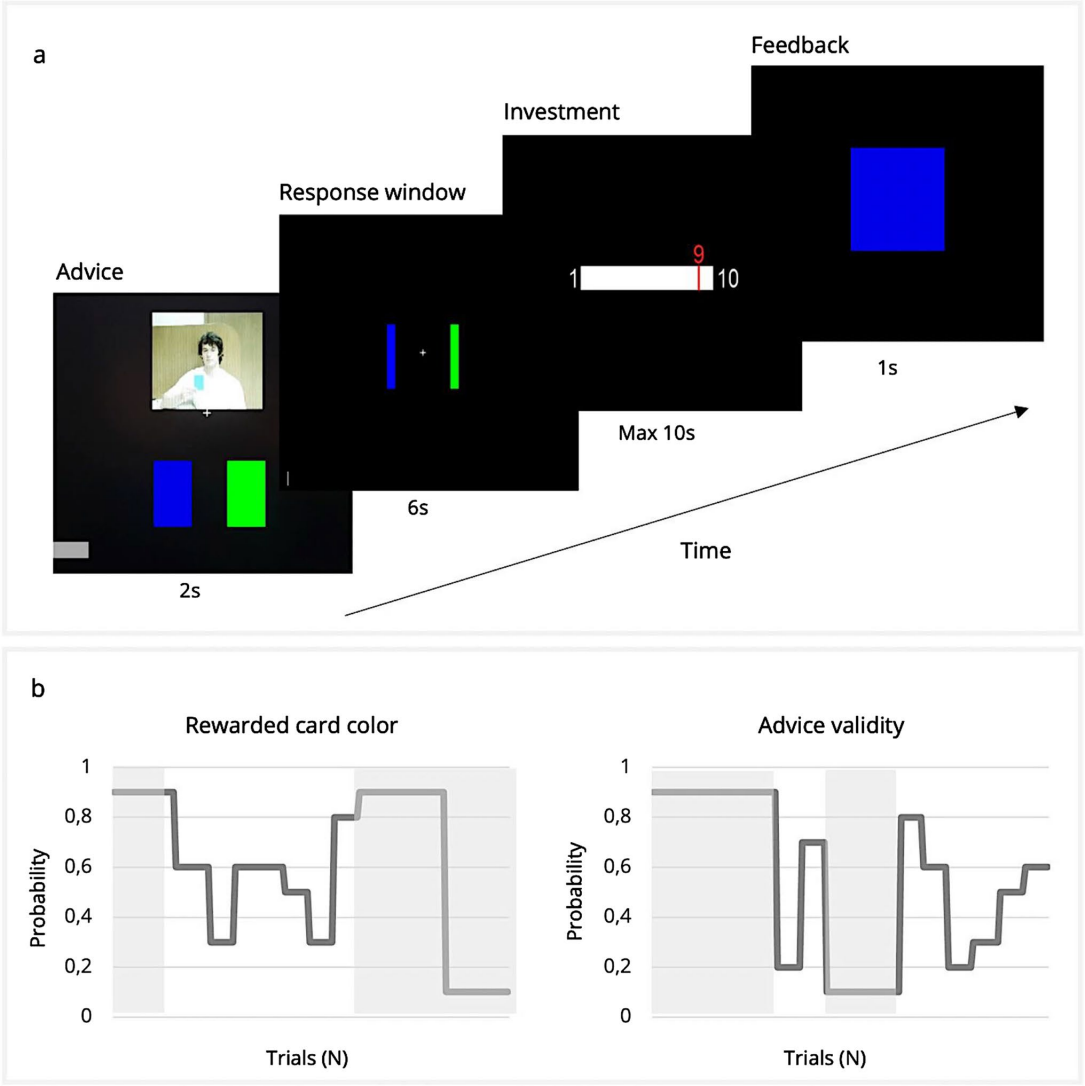
**a** Overview of the Wager task, adapted from Diaconescu et al., 2020**.** Each trial consisted of four phases: 1) Advice: advisor recommended a card; 2) Response: participant chose a card; 3) Investment: participant decided how many points to invest; and 4) Feedback: outcome was presented. **b** Probability scheme for the blue card being correct (top) and advisor trustworthiness (bottom) across trials. Stable phases are highlighted in light grey (Nonsocial: trials 1–25 and 100–160; Social: trials 1–49 and 70–99). Volatile phases are in grey (Nonsocial: trials 26–99; Social: trials 50–69 and 100–159). Adapted from Diaconescu et al. (2020)

The Wager task is a modified version of the deception-free binary lottery game created by Diaconescu and colleagues (2020) (Fig. 1a). The task included 160 trials with a short break after 70 trials. In each trial, participants were asked to predict the outcome of a card draw—blue or green—while they had access to two sources of information, information from the “advisor” (social information *or* social advice) and individually experienced recent outcome history (nonsocial information). In each trial, the draw was preceded by a short video clip that showed the advisor holding up one of the two cards, therewith recommending to the participant which card to choose. The video clips were previously recorded face-to-face sessions (Diaconescu et al., 2017). The advisors based their suggestion on true but probabilistic information with a constant probability of 80%; however, this was not made aware to the participant. Furthermore, the advisors received monetary incentives to change their strategy and provide either helpful or misleading advice at different phases of the task. Although the original study included four advisors (two males, two females), the current study used the video clips of one male advisor for all participants to reduce variance in the task. Diaconescu and colleagues (2017) showed that there were no performance differences or differences in reliance on the advice between different advisors in a similar task. In addition to the opportunity to infer the advisor’s trustworthiness, participants could infer the card probabilities based on the reward outcome history. They could infer the rewarding card color probabilities (blue vs. green) from outcomes of previous trials. Thus, in each trial, participants had to weigh two sources of information, social information (advice, inferring on intention) or individual information (previous rewarded card color, inferring on probability). The color-reward probabilities and the advisor trustworthiness varied independently across trials. This resulted in four different task phases: 1) stable nonsocial information and volatile social information; 2) stable nonsocial information and stable social information; 3) volatile nonsocial information and stable social information; and 4) volatile nonsocial information and volatile social information (Fig. 1b). The average probabilities were identical for both information sources, and the two sources were uncorrelated, as illustrated by phases of low (yellow) and high (light grey) volatility (Fig. 1b).

After the selection of one of the cards (blue or green), participants were asked to wager a number of points between one and ten to indicate how confident they were about their predictions. In each trial, the start position of the marker of the wager was random to ensure that participants did not wager points based on the previous trial. The score on each trial was dependent on the participant’s wagered number of points and, consequently, the participant’s payment at the end of the experiment. After the wager screen, the winning card (i.e., outcome) was presented. For each trial, the cumulative score was updated according to the won or loss wager of the previous trial and was presented as a bar at the bottom of the screen. A white bar indicated a positive total score, and a red bar indicated a negative total score. To increase the participants’ motivation, they were told that they could reach a silver (€2,50) and a golden (€5,00) bonus target. These targets were invisible during the task to avoid loss of motivation.

Furthermore, we asked participants to rate the usefulness of the advice based on a multiple-choice question (including the options: helpful, misleading, or neutral) that was presented at six random time points throughout the task. This allowed us to assess whether at any point in time, the model could significantly predict participants’ ratings of the advisor’s trustworthiness.

### Procedure

The present study was part of a larger project in which participants performed three different computer tasks (Driessen et al., 2021a, b). The order of the tasks was randomized between participants and tasks other than the Wager task are not considered in this article. Participants received instructions before the start of the task, and the experimenter checked whether each participant understood the instructions correctly. At the end of the experiment, participants were informed about the bonus amount they had earned and received their monetary compensation.

### Electrophysiological recordings

Electrophysiological data were collected using 32 active scalp electrodes (ActiCap, Brain Products, Munich, Germany) arranged according to a variation of the international 10–20 system, with an additional electrode on the right mastoid. Electrophysiological data was acquired at 500 Hz without filtering with the BrainAmp amplifier (Brain Products), with the reference electrode placed over the left mastoid bone with self-adhesive rings. In addition, six passive electrodes were used to collect horizontal and vertical eye movements and an electrocardiogram. Vertical eye movements were recorded by placing electrodes above and below the left eye, and horizontal eye movements were registered at the outer canthi of the eyes.

### Analyses

**Fig. 2:**
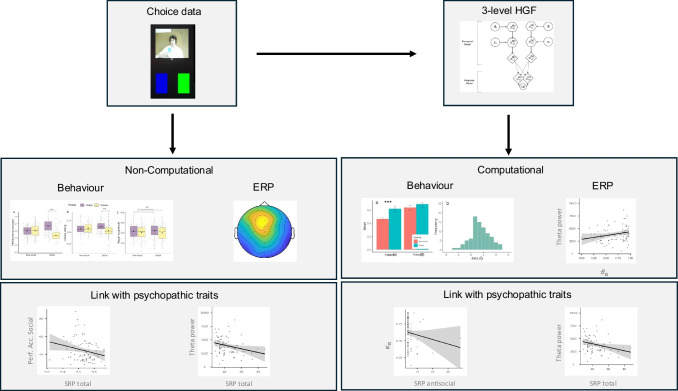
Overview of the analysis pipeline. Note that the correlations are Spearman rank correlations (1000 bootstrap samples)

A summary of the analytical pipeline is presented in Fig. 2.

#### Computational modelling of behavioral responses

**Fig. 3:**
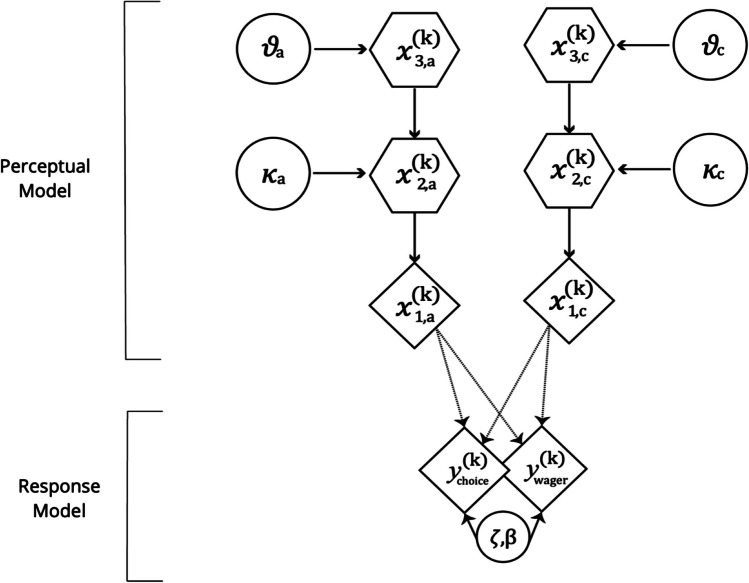
Model of the 3-level HGF and the response model. The diamonds represented quantities that change in time (i.e., that carry a time or trial index (k)) but that do not depend on their previous state. The hexagons represented states that change in time but additionally depend on their previous state. Circles denoted fixed parameters. The perceptual model had three layers: (1) χ1 represented the accuracy of the current advice or the rewarded card color; (2) ${\upchi }_{2}^{(\text{k})}$ represented the perceived likelihood of obtaining trustworthy advice or of the card-outcome contingency on trial (k); and (3) ${\upchi }_{3}^{(\text{k})}$ represented the participant’s representation of the rate of change in the advisor’s trustworthiness or the card-outcome contingency on trial (k). Parameter $\kappa$ captures how strongly ${\upchi }_{2}^{(\text{k})}$ and ${\upchi }_{3}^{(\text{k})}$ are coupled, and $\vartheta$ indicates the estimated rate of change in ${\upchi }_{3}^{(\text{k})}$. The response model had two layers: 1) the computation of the probability of the outcome given both the nonsocial and the social source; 2) the chosen action. Parameter $\zeta$ reflected the weight of the social- compared to the nonsocial source. $Y$ represents the subject’s binary response ($y$= 1: responding according to the advice, $y$ = 0: responding against the advice). The results on the inverse decision temperature parameter beta ($\beta$) were not considered in the current study. Figure adapted from Diaconescu et al., (2017, 2020)

In the present study, we examined how subjects updated their representations about others’ trustworthiness and chose to follow or disregard their advice. For this purpose, we applied a computational model which is referred to as the Hierarchical Gaussian Filter (HGF), a generic Bayesian model of learning under perceptual and environmental uncertainty that contextualizes RL within a generic Bayesian scheme and thus connects it to principles of optimality from probability theory (Mathys et al., 2011, 201,4). HGF models have been widely used for analyses of time-series data in the behavioral, psychophysiological and brain domains (Brazil et al., 2017; De Berker et al., 2016; Hauser et al., 2014; Marshall et al., 2016; Vossel et al., 2014). Based on previous research on social- and reward-based learning mechanisms, we opted to employ a 3-level HGF model that assumes that participants learn according to a mechanism in which they track the rate of change and the overall volatility of both social and nonsocial information (Diaconescu et al., 2014, 2017, 2020). This model was previously used to examine arbitration between reward and socially-relevant information (Diaconescu et al., 2020). Based on this model, we assumed that the rewarded card colour (nonsocial learning) and the advice accuracy (social learning) varied as a function of hierarchically coupled hidden states ${\upchi }_{1}^{(\text{k})}$, ${\upchi }_{2}^{(\text{k})}$,… ${\upchi }_{\text{n}}^{(\text{k})}$. They evolved in time by performing Gaussian random walks. At any given level, the step size was controlled by the state of the next-higher level (Fig. 3). The lower-level hierarchy states ${\upchi }_{1\text{a}}^{(\text{k})}$ and ${\upchi }_{1\text{c}}^{(\text{k})}$ were binary and represented the advice accuracy (1 for accurate, 0 for inaccurate) and the rewarded card colour (1 for blue, 0 for green). The second level hierarchy states ${\upchi }_{2\text{a}}^{(\text{k})}$ and ${\upchi }_{2\text{c}}^{(\text{k})}$ were continuous variables and represented the advisor’s fidelity and the tendency for a given card colour to be rewarding. The third level hierarchy states ${\upchi }_{3\text{a}}^{(\text{k})}$ and ${\upchi }_{3\text{c}}^{(\text{k})}$ were also continuous and denoted the rate of change of the advisor’s trustworthiness and the card-outcome contingencies. Four learning parameters, κ_*a*_, κ_*c*_, ϑ_*a*_, and ϑ_*c*_, determined the rate with which the hidden states evolved in time. The parameters κ_*a*_ and κ_*c*_ determined the coupling between the second and third hierarchy levels, whereas ϑ_*a*_ and ϑ_*c*_ determined the variance of the volatility (i.e., meta-volatility) over time. In agreement with Bayes’ rule, we assumed that participants who made inferences based on advice and card outcomes generated representations about advice-outcome congruency and the rewarding card colour. The application of the Bayes’ rule to our generative model (i.e., model inversion) results in a “perceptual model,” capturing participants’ representations concerning volatility.

As in the previous study (Diaconescu et al., 2020), the “response model” maps the predicted outcomes to behavioural responses (blue or green card) and predicts two components of the outcome on each trial: 1) the participant’s decision to accept ($y$= 1) or reject ($y$= 0) the advice, and 2) the number of points wagered. We assumed that a prediction of the outcome on a given trial is defined as a function of the perceived reliability of each information source (i.e., nonsocial vs. social) and the predictions afforded by each source. This arbitration was captured with the response model parameter ζ, which represents the participant’s bias towards social information, and has a prior mean of 1, indicating an equal weighing of the two information sources. A value of ζ > 1 indicates a bias towards advice, while a value of ζ < 1 indicates a bias towards the estimated card colour probability. The beta parameter (*β*) represents the inverse decision temperature and determines the belief-to-response mapping (i.e., does the participant behave according to their prediction of the outcome probabilities). This parameter was set to vary, but the results were not considered in the current study. The formal equations underlying the model and the model inversion are described in the studies of Diaconescu and colleagues (2014, 2020). An overview of how the parameters map onto psychological mechanisms can be found in the Supplemental material (Table S5).

#### Wavelet analysis

Electrophysiological data was analyzed using MATLAB (v2015b, MathWorks Inc., Natrick, MA), in combination with the Fieldtrip toolbox (Oostenveld et al., 2011). Data were filtered offline by using a 0.01–40-Hz filter and re-referenced to the average of the linked mastoids. Manual artifact rejection was conducted to detect muscle and electrode artifacts in the data. Subsequently, EOG and ECG artifacts were removed using Independent Component Analysis (ICA) in the Fieldtrip toolbox. With the use of ICA, components that contained ocular artifacts were identified by inspecting the time course and spatial topography of all components. For each participant, activity associated with the feedback cue was extracted for each trial in epochs starting 500 ms prior to the stimulus onset and ending 1500 ms after stimulus onset. Next, time–frequency representations (TFRs) were calculated by using Morlet wavelets with a width of 3 (Tallon-Baudry and Bertrand, 1999), in 40 steps within the frequency range of 1–40 Hz, separately for different conditions (i.e., all trials, correct trials only, incorrect only, stable nonsocial information, volatile nonsocial information, stable social information, and volatile social information). Subsequently, mean theta power (4–8 Hz) over the midfrontal electrodes Fz, FCz, and Cz were calculated and extracted for the time window of 100–400 ms after feedback presentation.

#### Statistical analyses

##### Behavioural data analysis

To test for the significant effects of phase (stable, volatile) and source (nonsocial information, social information) on performance accuracy (i.e., the number of points earned depending on selecting the correct card and the number of points wagered), advice taking, and wager magnitude, general linear models (GLMs) for repeated measures with phase and source as within-subject measures were used (two-tailed). Effects of phase and source on the computational learning model parameters *κ* and *ϑ* for both social and nonsocial information were tested by using a repeated measures GLM with both phase and source as within-subject measures. Furthermore, a one-sample *t*-test was performed to investigate whether ζ was different from one. Finally, the link between performance accuracy, advice-taking, wager magnitude, and the computational learning parameters and the SRP total and subscale scores were tested using Spearman correlations.

##### Computational model selection

We conducted a model comparison using a Rescorla-Wagner model, a Kalman Filter, a 2-level Hierarchical Gaussian Filter (HGF) model, and a 3-level HGF model. Unlike the 2-level model, the 3-level HGF model assumes that participants track the volatility of both information sources. All models were fitted using the HGF toolbox and the models were compared using Bayesian model selection (BMS) (Stephan et al., 2009), as implemented in the Statistical Parametric Mapping 12 toolbox (https://www.fil.ion.ucl.ac.uk/spm/). We compared the models based on Bayesian Information Criteria (BIC) and Log Model Evidence, which represent a trade-off between model complexity and model fit.

##### Internal validity test of the winning model

To test the internal validity of the 3-level HGF model, a linear regression analysis was performed to examine whether the model predictions were consistent with participant’s ratings of advisor’s trustworthiness during the experiment. Furthermore, Spearman correlations (1000 bootstrap samples) were conducted to investigate whether the wager magnitudes predicted by the model (i.e., predicted wagers) significantly correlated with the participant’s actual wagers.

##### Parameter recovery checks

To ensure the model was a realistic model of the data, we conducted 100 simulations per participant using individual parameter estimates from the model. Individuals’ binary choices were averaged on a trial-wise basis to estimate if the behaviour simulated from the parameters matched the true behaviour (average win-stay and lose-shift rates). Moreover, the simulated and the true choice trajectory, as well as the simulated and true wager amounts per trial were compared. Lastly, we fit the perceptual and response model to the simulated data by choosing 10 of the simulations at random and recovered their parameters. Spearman correlations (9999 bootstrap samples) were reported (Bayesian zero-order correlations can be found in the Supplemental material).

##### ERP data analyses

To test for the significant effects of phase and source on mean theta power over the midfrontal electrodes (mean of Fz, FCz, and Cz; for short, “theta power”), a GLM for repeated measures with phase and source as within-subject variables were used. A paired *t*-test was used to study a potential difference between theta power over correct and incorrect trials. Moreover, Spearman correlations were conducted to examine the link between theta power and the overall accuracy, the accuracy over volatile and stable trials, and the computational parameters.

##### Correlational analyses

Lastly, Spearman correlations (1000 bootstrap samples) were tested to study the link between psychopathic personality traits and midfrontal theta and theta power over individual electrodes (Fz, FCz, Cz). Interpretation of the results were based on bootstrapped confidence intervals. In addition to the frequentist statistics, we performed correlations using Bayesian statistics as an alternative approach. We considered an effect to be strongly supported only when both analytical approaches yielded a similar result (for a similar approach see studies of Driessen et al. (2018) or Maes & Brazil (2015)). The results of these Bayesian correlations can be found in the Supplemental material (Tables S1− 3).

## Results

### Behavioral results

#### Performance metrics: performance accuracy, advice-taking and wager magnitude

**Fig. 4:**
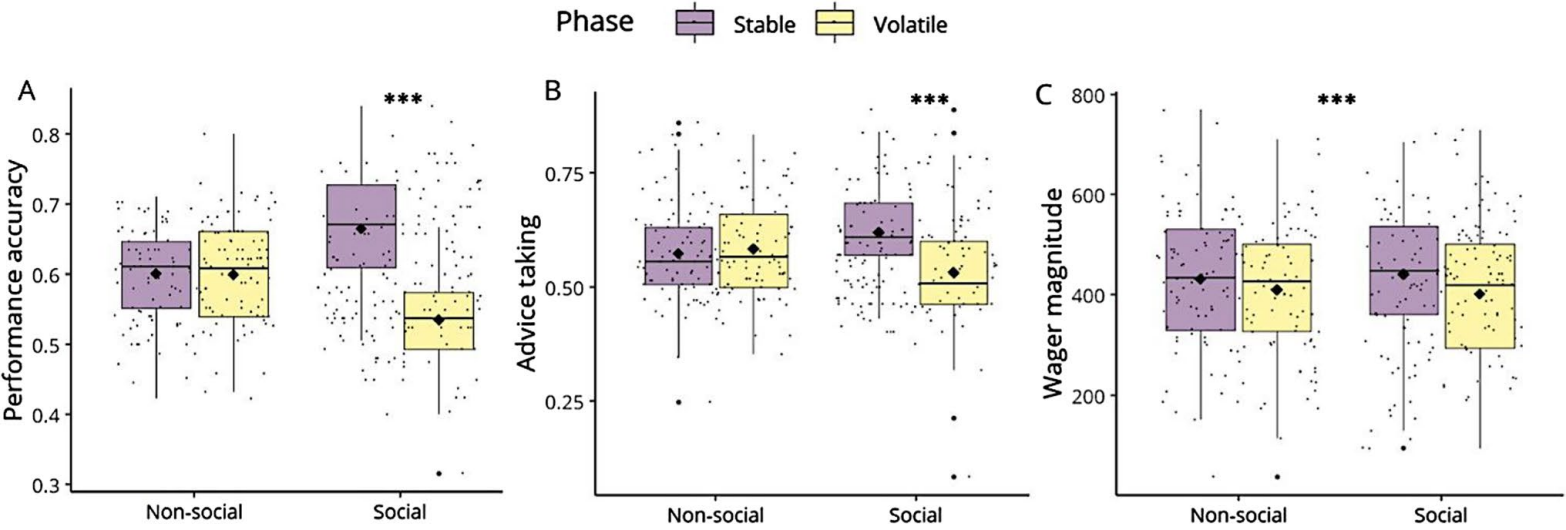
Behavioural variables influenced by the volatility. **A** Performance accuracy; **B** advice-taking; and **C** amounts of points wagered. Black diamonds represent the means. Significant effects are flagged (***p <.001). **A** Participants performed significantly better in phases of the task in which social information was stable as compared to when social information was volatile (*t*(85) = 11.197, *p* <.001, *d* = 1.21, 95% CI [.93, 1.48]). **B** Participants took advice into account more often in phases of the task in which social information was stable compared with when social information was volatile (*t*(85) = 6.062, *p* <.001, *d* =.654, 95% CI [.42,.89]). **C** Participants wagered significantly more points during stable phases of the task (*F*(1,85) = 98.409, *p* <.001, η_p_^2^ = *.*537, 95% CI [.39,.64]), regardless of the information source

Figure 4 shows the descriptive results for performance accuracy, advice taking, and reward magnitude. Overall performance accuracy was 60.1% ± 4.1% (mean ± standard deviation). There was a main effect of phase (*F*(1,85) = 358.314, *p* < 0.001, η_p_^2^ = 0.808, 95% confidence interval [CI] [0.73, 0.85]), and a source x phase interaction (*F*(1,85) = 38.017, *p* < 0.001, η_p_^2^ = 0.309, 95% CI [0.15, 0.44]). Post hoc analyses revealed that participants performed significantly better in phases of the task in which social information was stable as compared to when social information was volatile (*t*(85) = 11.197, *p* < 0.001, *d* = 1.21, 95% CI [0.93, 1.48]), while there was no phase-dependent difference for learning from nonsocial information (*t*(85) = 0.163, *p* = 0.871,* d* = 0.018, 95% CI [− 0.19, 0.23]). This indicated that the degree to which participants relied on the social information was depended on the volatility structure of the task (Fig. 4a). There was no significant main effect of source on performance accuracy (*F*(1,85) = 0.189, *p* = 0.665, η_p_^2^ = 0.002, 95% CI [0, 0.06]).

Participants took advice in 57.6% ± 9.0% of the trials. There was also a main effect of phase (*F*(1,85) = 41.884, *p* < 0.001, η_p_^2^ = 0.330, 95% CI [0.17, 0.46]) and a source x phase interaction (*F*(1,85) = 17.741, *p* < 0.001, η_p_^2^ = 0.173, 95% CI [0.50, 0.31]) on advice-taking. Post-hoc analyses showed that participants took advice into account more often in phases of the task in which social information was stable as compared to when social information was volatile (*t*(85) = 6.062, *p* < 0.001, *d* = 0.654, 95% CI [0.42, 0.89]). Phase of nonsocial information did not have a significant effect on advice-taking (*t*(85) = − 0.880, *p* = 0.382, − 0.095, 95% CI [− 0.31, 0.11]) (Fig. 4b). There was no significant main effect of information source on advice-taking (*F*(1,85) = 1.190, *p* = 0.278, η_p_^2^ = 0.014, 95% CI [0, 0.01]).

Finally, the overall average points wagered was 5.38 ± 1.53. A main effect of phase on the amount of points wagered was observed (*F*(1,85) = 98.409, *p* < 0.001, η_p_^2^ = *0.5*37, 95% CI [0.39, 0.64]). This indicated that, regardless of the type of information source, participants wagered significantly more points during stable phases of the task (i.e., where at least one source of information was stable) as compared to volatile phases (Fig. 4c). There was no significant main effect of source (*F*(1,85) < 0.001, *p* = 1.000, η_p_^2^ < 0.001, 95% CI [0, < 0.001]) and no significant interaction effect of phase x source (*F*(1,85) = 1.942, *p* = 0.169, η_p_^2^ = 0.022, 95% CI [0, 0.12]) on the amount of points wagered.

#### Performance metrics and psychopathic traits

Performance accuracy during volatile phases of the task appeared to be associated with psychopathic traits. Accuracy during volatile trials was negatively correlated with lifestyle traits (ρ = − 0.221, *p* = 0.041, 95% CI [− 0.43, − 0.01]), and antisocial traits (ρ = − 0.232, *p* = 0.032, 95% CI [− 0.41, − 0.02]). This indicated that individuals with higher levels of lifestyle-antisocial traits were affected by volatility. More specifically, the association between psychopathic traits and accuracy for volatile trials was driven by the volatile social trials (total score: ρ = − 0.235, *p* = 0.029, 95% CI [− 0.43, − 0.03], interpersonal: ρ = − 0.228, *p* = 0.035, 95% CI [− 0.42, − 0.02]; affective: ρ = − 0.253, *p* = 0.019, 95% CI [− 0.44, − 0.04]; antisocial: ρ = − 0.231, *p* = 0.032, 95% CI [− 0.41, − 0.02]), while the effect was not present in the volatile nonsocial trials (Table 1; Bayesian correlations can be found in Table S1). Psychopathic traits were not associated with the degree of advice-taking (total score: ρ = 0.019, *p* = 0.862, 95% CI [− 0.20, 0.24]) or the number of points that was wagered (total score: ρ = − 0.035, *p* = 0.752, 95% CI [− 0.19, 0.23]) on the different trial conditions.

**Table 1:** Spearman’s correlations between SRP scores and performance accuracy

	Total score	Interpersonal	Affective	Lifestyle	Antisocial
	*rho,* 95% CI	*rho,* 95% CI	*rho,* 95% CI	*rho,* 95% CI	*rho,* 95% CI
Perf. acc					
Overall	−.178 [−.38,.06]	−.152 [−.38,.09]	−.133 [−.35,.12]	−.209 [−.40, <.01]	−.144 [−.35,.08]
Stable	−.152 [−.36,.08]	−.124 [−.33,.10]	−.109 [−.34,.15]	−.188 [−.38,.02]	−.078 [−.29,.16]
Volatile	−.205 [−.40,.03]	−.187 [−.38,.03]	−.154 [−.36,.07]	−.221* [−.43, −.01]	−.232* [−.41, −.02]
Nonsocial info					
Stable	−.174 [−.35,.04]	−.135 [−.34,.10]	−.189 [−.38,.02]	−.149 [−.34,.04]	−.108 [−.29,.09]
Volatile	−.124 [−.35,.10]	−.120 [−.34,.10]	−.045 [−.28,.19]	−.173 [−.38,.04]	−.141 [−.35,.08]
Social info					
Stable	−.094 [−.31,.14]	−.078 [−.30,.15]	−.035 [−.29,.21]	−.151 [−.37,.05]	−.047 [−.28,.18]
Volatile	−.235* [−.43, −.03]	−.228* [−.42, −.02]	−.253* [−.44, −.04]	−.155 [−.38,.06]	−.231* [−.41, −.02]

Asterisks indicate significant correlations (*p* <.05). *CI* confidence interval (1000 bootstrap samples)

### Computational results

#### Model comparison

We could not achieve an adequate model fit of the data to the Kalman Filter model (posteriors centered on the prior for all subjects), indicating it was not a realistic model of the underlying behavior. Thus, we excluded it from the BMS procedure. The results indicated that the HGF models (2-level HGF: BIC = 914.48, LME = − 449.47; 3-level HGF: BIC = 917.11, LME = − 450.84) outperformed the Rescorla-Wagner model (BIC = 8792.53, LME = − 4386.34). For more details, please check the supplemental material. While the 2-level and 3-level HGF models demonstrated similar fits, the 3-level HGF performed slightly better. Given our hypotheses, we chose to use the 3-level HGF model for further analyses.

#### Parameter recovery checks

A comparison of the behaviour simulated from the parameters and the true behaviour demonstrated a good match. First, the simulated and original win-stay rates and lose-shift rates were positively correlated (win-stay: ρ = 0.47, 95% CI [0.27, 0.64]; lose-shift: ρ = 0.61, 95% CI [0.44, 0.74]) The simulated and original wager amounts were also positively correlated (ρ = 0.99, 95% CI [0.98, 1.00]). Second, a visual inspection of the simulated and the true choice trajectory indicates that the model accurately captures the original behavior (see also Fig. S1 in the Supplemental material). The correlations between the trial-wise choices (averaged across participants) were ρ = 0.79, [0.71, 0.85], further supporting this finding. Third, a comparison between the simulated and true wager amounts per trial demonstrated that, in general, the pattern is preserved (ρ = 0.46, [0.31, 0.57]) (see also Fig. S2 in the Supplemental material). However, note that the model tends to simulate more conversative wagers than the participants actually made (e.g., if a participant wagered 9, the model would estimate they wagered 4–5, and generally never go above this amount). Lastly, we fit the perceptual and response model to the *simulated* data and recovered their parameters. The recovered parameters adequately match the originally estimated parameters (κ_*c*_: ρ = 0.39, 95% CI [0.19, 0.57]; κ_*a*_: ρ = 0.76, 95% CI [0.64, 0.85], ϑ_*c*_: ρ = 0.30, 95% CI [0.09, 0.49]; ϑ_*a*_: ρ = 0.51, 95% CI [0.30, 0.67]; ζ: ρ = 0.93, 95% CI [0.90, 0.96]). Bayesian zero-order correlations were reported in the Supplemental material Tables S7 and S8.

#### Model’s internal validity

**Fig. 5:**
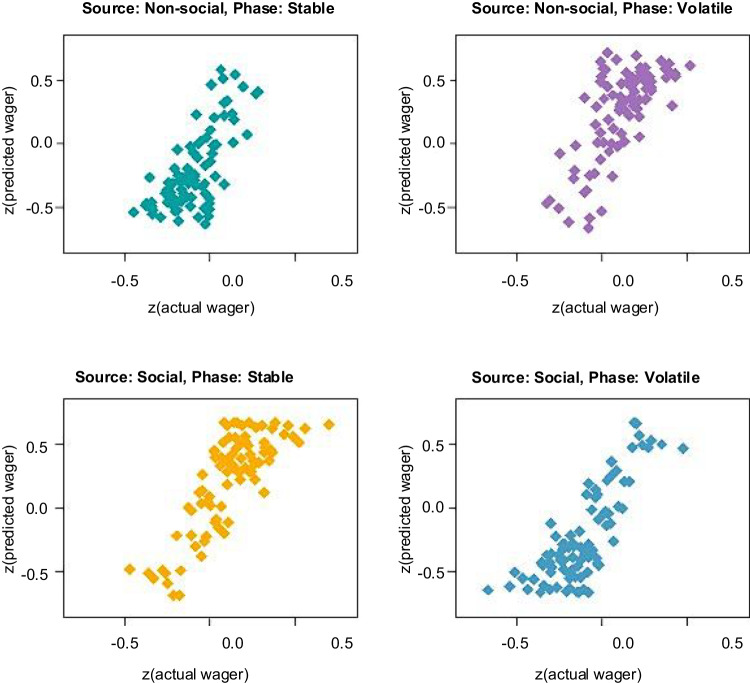
Model internal validity. The predicted wager was highly correlated with the number of points participants actually wagered across all four phases of the experiment

As an internal validity check, we examined whether the model predictions were consistent with participant’s ratings of the advisor’s trustworthiness during the experiment. Participant’s estimates of advice accuracy extracted from the model reflected the reported advisor fidelity on the randomly presented multiple choice questions (including the response options: helpful, misleading, neutral). A linear regression analysis showed that participants’ advisor ratings were predicted by their estimated advisor accuracy at the time of the presentation of the multiple-choice question. In addition, the wager magnitudes predicted by the model significantly correlated with the participants’ actual wagers. Across the four phases of the task, the predicted wager was highly correlated with the number of points participants actually wagered: stable social information ρ = 0.764, *p* < 0.001, 95% CI [0.66, 0.84]; volatile social information ρ = 0.768, *p* < 0.001, 95% CI [0.67, 0.84]; stable nonsocial information ρ = 0.640, *p* < 0.001, 95% CI [0.50, 0.75]; volatile nonsocial information ρ = 0.637, *p* < 0.001, 95% CI [0.49, 0.47]) (Fig. 5).

#### Learning parameter estimates

**Fig. 6:**
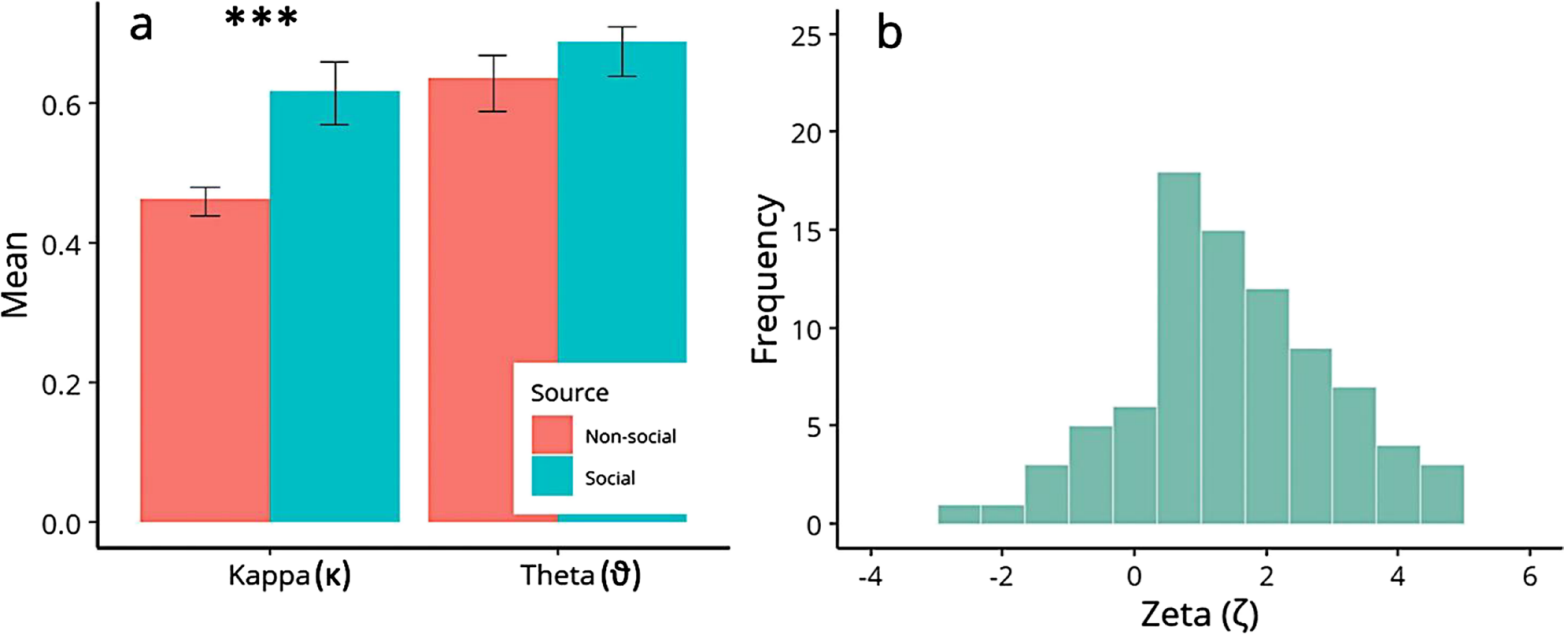
**a** Learning model parameters. Results showed that there was a significant difference between kappa ($\kappa$) for social information and kappa ($\kappa$) for nonsocial information. This was not the case for theta. **b** Distribution of values for response model parameter zeta ($\zeta$)

While there was no significant difference in the meta-volatility parameter $\vartheta$ across the two different learning domains, the coupling parameter $\kappa$ was significantly different as a function of information source (*F*(1,85) = 42.914, *p* < 0.001, η_p_^2^ = 0.335, 95% CI [0.18, 0.47]) (Fig. 6a). This result suggests that there was a difference in how participants learned from volatile reward probabilities and advisor trustworthiness, with stronger coupling for nonsocial information as compared to social information (${\upkappa }_{c}$ = 0.62, ${\upkappa }_{a}$ = 0.46). Response model parameter $\zeta$, reflecting the level of social bias, was significantly different from zero (*t*(85) = 7.223, *p* < 0.001, *d* = 0.779, 95% CI [0.54, 1.01]) (Fig. 6b). This indicates that, on average, participants preferred relying on the social over nonsocial information when making decisions.

#### Learning parameters and psychopathic traits

The coupling parameter ${\kappa }_{a}$ was significantly associated with antisocial traits (ρ = − 0.235, *p* = 0.029, 95% CI [− 0.43, − 0.02]), which indicated that the information flow between ${\upchi }_{2}^{(\text{k})}$ and ${\upchi }_{3}^{(\text{k})}$ for social information was reduced with increasing levels of antisocial traits. This was not the case for nonsocial information (ρ = 0.176, *p* = 0.106, 95% CI [− 0.01, 0.35]). Furthermore, meta-volatility parameters${\vartheta }_{a}$,${\vartheta }_{c}$, and the social bias parameter $\zeta$ were not associated with psychopathic traits (${\vartheta }_{a}$ – total score: ρ = − 0.100, *p* = 0.361, 95% CI [− 0.30, 0.11]; ${\vartheta }_{c}$ – total score: ρ = 0.077, *p* = 0.479, 95% CI [− 0.13, 0.29]; $\zeta$ – total score: ρ = 0.023, *p* = 0.830, 95% CI [− 0.19, 0.25]).

### EEG results

**Fig. 7:**
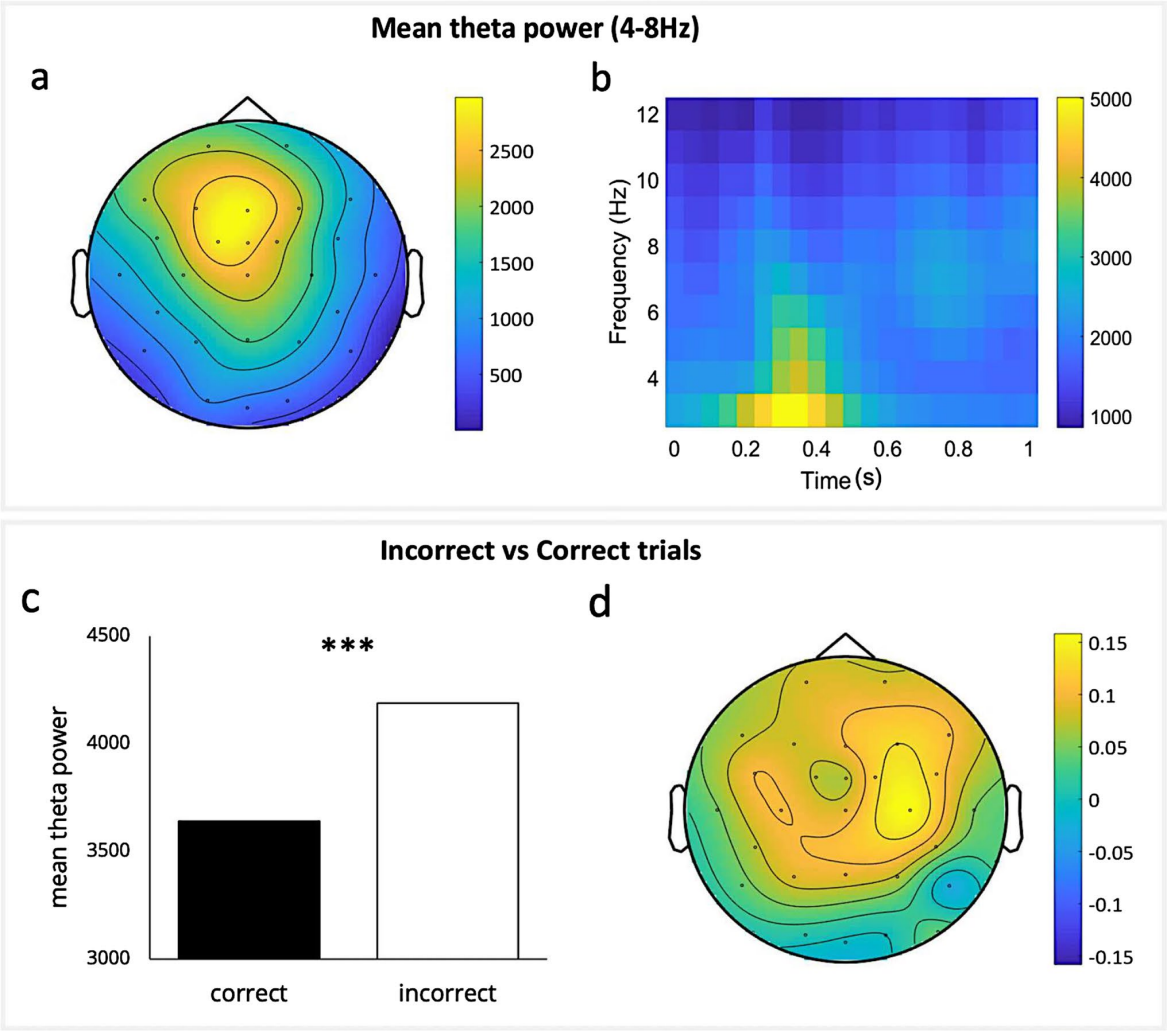
Mean theta power (4–8 Hz). **a** Topoplot of mean theta power across 100–400 ms after trial outcome representation (0 ms). **b** Time–frequency plot of mean theta power (4–8 Hz) over the midfrontal electrodes (Fz, FCz, Cz) across 0–1 s after trial outcome representation. **c** Mean theta power over the midfrontal electrodes for correct and incorrect trials. **d** Topographic representation of the TFR of the difference in theta power between incorrect and correct trials

Figure 7a shows a topoplot and Fig. 7b a time–frequency plot of mean theta power over the midfrontal electrodes (Fz, FCz, Cz) across 100–400 ms after trial outcome representation. We did not find main effects (source: *F*(1,77) = 0.116, *p* = 0.734; η_p_^2^ = 0.002, 95% CI [0, 0.54]; phase: *F*(1,77) < 0.001, *p* = 0.998; η_p_^2^ < 0.001, 95% CI [0, < 0.001]), or an interaction effect for source and phase (*F*(1,77) = 3.339, *p* = 0.069; η_p_^2^ = 0.042, 95% CI [0, 0.14]). Performance accuracy was positively associated with midfrontal theta power (ρ = 0.231, *p* = 0.039, 95% CI [< 0.01, 0.41]). More specifically, accuracy over volatile trials was positively associated with midfrontal theta power on these trials (ρ = 0.294, *p* = 0.008, 95% CI [0.10, 0.47]). Accuracy over stable trials was not associated with midfrontal theta (Table 2; Bayesian correlations can be found in Table S2). Furthermore, theta power over incorrect trials was higher as compared to correct trials (*t*(79) = − 6.482, *p* < 0.001, *d* = − 0.717, 95% CI [− 0.96, − 0.47]) (Fig. 7c and d).

**Table 2:** Spearman’s correlations between performance accuracy and computational parameters and mean theta power over the midfrontal and individual electrodes

	Fz	FCz	Cz	Midfrontal
	*rho,* 95% CI	*rho,* 95% CI	*rho,* 95% CI	*rho,* 95% CI
Performance accuracy				
Overall	.181 [−.06,.37]	.245* [.01,.42]	.251* [.03,.43]	.231* [<.01,.41]
Volatile	.239*, [.01,.43]	.307** [.10,.48]	.314** [.09,.52]	.294** [.11,.49]
Stable	.113 [−.13,.31]	.175 [−.06,.36]	.179 [−.05,.37]	.159 [−.08,.35]
Nonsocial information				
Kappa $({\kappa }_{c}$)	−.004 [−.21,.21]	−.010 [−.23,.22]	−.061 [−.27,.18]	−.026 [−.25,.20]
Theta (${\vartheta }_{c})$	.027 [−.20,.25]	.026 [−.21,.26]	.010 [−.22,.24]	.014 [−.22,.24]
Social information				
Kappa (${\kappa }_{a})$	.133 [−.06,.34]	.217 [.03,.40]	.248* [.06,.44]	.202 [.02,.39]
Theta (${\vartheta }_{a}$)	.156 [−.05,.37]	.232* [.04,.43]	.271* [.08,.46]	.221* [.03,.42]

Asterisks indicate significant correlations (*p <.05, **p <.01). *CI* confidence interval (1000 bootstrap samples); Perf. acc = Performance accuracy; info. = information

Analyses to investigate a possible association between theta power and the computational parameters showed us that the meta-volatility parameter ${\vartheta }_{a}$ for social information positively correlated with theta power (ρ = 0.221, *p* = 0.049, 95% CI [< 0.001, 0.40]). The other parameters did not show a significant correlation with theta power (Table 2; Bayesian correlations can be found in Table S2). Furthermore, we were interested in a possible link between psychopathic personality traits and mean theta power. SRP total scores were negatively correlated with theta power over the midfrontal electrodes (ρ = − 0.385, *p* < 0.001, 95% CI [− 0.56, − 0.15]), as well as the interpersonal (ρ = − 0.353, *p* = 0.002, 95% CI [− 0.56, − 0.14]), affective (ρ = − 0.366, *p* < 0.001, 95%CI [− 0.54, − 0.15]), lifestyle (ρ = − 0.322, *p* = 0.004, 95% CI [− 0.52, − 0.11]), and antisocial (ρ = − 0.364, *p* < 0.001, 95% CI [− 0.52, − 0.10]) facet scores (Table 3; Bayesian correlations can be found in Table S3).

**Table 3:** Spearman’s correlations between SRP scores and mean theta power over the midfrontal and individual electrodes

	Fz	FCz	Cz	midfrontal
	*rho,* 95% CI	*rho,* 95% CI	*rho,* 95% CI	*rho,* 95% CI
Interpersonal	−.308* [−.50, −.08]	−.355** [−.53, −.12]	−.345** [−.52, −.11]	−.353** [−.56, −.14]
Affective	−.286* [−.47, −.06]	−.371*** [−.55, −.12]	−.386*** [−.52, −.11]	−.366*** [−.54, −.15]
Lifestyle	−.270* [−.48, −.04]	−.325** [−.52, −.11]	−.325** [−.52, −.10]	−.322** [−.52, −.11]
Antisocial	−.283* [−.46, −.07]	−.372*** [−.52, −.11]	−.391*** [−.52, −.10]	−.364*** [−.52, −.10]
SRP total	−.316** [−.50 −.07]	−.391** [−.56, −.16]	−.396*** [−.56, −.17]	−.385*** [−.56, −.15]

Asterisks indicate significant correlations (*p <.05, **p <.01, ***p <.001). *CI* confidence interval (1000 bootstrap samples)

## Discussion

The current study demonstrated that elevations in levels of psychopathic traits are accompanied by less associative learning from social information. Moreover, it showed that antisocial traits are linked to a reduced ability to adapt to changes in the reliability of social information. These alterations did not relate to a preference for one of the information sources over the other, and task performance did not correlate with the risk that was taken to obtain a high reward (i.e., the number of coins wagered). Furthermore, it was found that decreased theta activity was linked to higher levels of psychopathic traits in general, which aligns with indications that theta activity is involved in tracking the volatility of social information. In the following paragraphs, we will first discuss the findings for the general, noncomputational, behavioral performance measures and their relationship with psychopathic traits in detail. Then, we will consider the novel insights brought by the results obtained using the computational model and the electrophysiological measures, and how they may advance our understanding of psychopathy.

Our noncomputational findings showed that participants performed better and took social information more into account during stable phases of the task as compared to volatile phases. Moreover, participants wagered more points during stable phases of the task, independent of whether the information was social or nonsocial. These findings demonstrated the effectiveness of the manipulation of volatility in our task. Regarding psychopathy, the results suggested that increases in scores on erratic lifestyle and antisocial traits were associated with deteriorated performance during volatile phases of the task. This could be explained by considering the indications that excessive behavioral activation is a key component underlying the erratic lifestyle and antisocial facets. Previous studies have proposed that incarcerated individuals with relatively high levels of lifestyle-antisocial psychopathic traits are characterized by a tendency to excessively pursue appetitive stimuli. This tendency has been associated with a decreased ability to properly monitor and adjust ongoing behavior in response to changes in environmental contingencies (Buckholtz et al., 2010; Fowles, 1980; Hoppenbrouwers et al., 2015; Newman et al., 1987, 2017, 2005). The association between volatility and psychopathic traits was statistically significant for social information only, which is consistent with the proposed impairments in social learning and behavior in psychopathy (Blair, 2007; Brazil et al., 2011, 2013a). Finally, the number of points wagered on each trial, reflecting the level of confidence about the choice made, was unrelated to psychopathic traits. The latter result could be seen as an indication that these individuals remained confident that they were making the right choices, despite their relatively poor performance. Previous findings showed that monitoring of one’s own behavior is not completely affected in psychopathic offenders (Brazil et al., 2009, 2011; Steele et al., 2016), which suggests that poor performance monitoring may not provide the most suitable explanation in this case, especially in the less extreme population included in the present study. The observed overconfidence in decision-making abilities does align with prior reports suggesting an increased behavioral activation towards appetitive stimuli in individuals with high scores on the lifestyle-antisocial component of psychopathy. The excessive urge to pursue reward is associated with an insensitivity to inhibitory cues and blame externalization, which may explain overconfidence and risky decision-making. However, the present findings suggest that an excessive tendency to pursue reward is not restricted to lifestyle-antisocial traits, but is related to the other facets as well. Besides, a recent study of Atanassova et al. (2025) found that individuals scoring high on antisocial traits tend to have a biased belief that learned stimulus-outcome associations change rapidly, especially in situations were high saliency rewards were present. As a result, unexpected outcomes are perceived as less surprising, resulting in diminished learning and reduced behavioural adaptation (i.e., lower performance).

While informative, these results do not elucidate how psychopathy might be related to disturbances in the various interacting cognitive processes that operate within the associative learning mechanism. Therefore, a computational model was used to untangle and quantify the latent cognitive computations that underlie the behavioral patterns and performance of each participant. We found a difference in the coupling parameter capturing the flow of information between processes involved in estimating the rate of change and overall changeability of the advisor’s trustworthiness in providing helpful or misleading advice, respectively. This indicates that, in general, it is more difficult to estimate the likelihood and the rate of change for social advice than for nonsocial information. During nonsocial learning, the individual can rely on outcome information that is relatively easy to identify (e.g., a coin indicating reward), while a social context introduces additional levels of complexity that requires the involvement of higher-order cognitive processes to determine the valence of the outcomes (e.g., making inferences about other’s mental states) (Buades-Rotger et al., 2023; Lee & Harris, 2013). We suggest that stronger coupling between the two levels of the computational hierarchical in a social context result from an increased sense of uncertainty about the fidelity of social information. That is, the use of nonsocial information requires an individual to rely predominantly on her/his own estimates of the card probabilities and their rate of change, while using social information requires a stronger dependency on the trustworthiness of the advisor: individuals may experience more control during the former as compared to the latter (Collins et al., 2011; Diaconescu et al., 2014; Van’t Wout, & Sanfey, 2008).

Regarding psychopathic traits, the findings point out that the interaction between the estimated likelihood and the estimated rate of change of social information was reduced (i.e., kappa values were lower) with increasing levels of antisocial traits. One possible explanation is that individuals with elevated levels of antisocial traits do not sufficiently process the complexity of the social context. That is, some cognitive processes that are required for proper evaluation of the social context may be less engaged (Brazil et al., 2011, 2013a), resulting in a smaller need to update representations concerning the volatility of social advice. The lower values of the coupling parameter kappa during social inference could reflect this relative ‘disengagement’ of the mechanism in this regard. This explanation aligns with our other behavioral findings indicating a negative relationship between the level of psychopathic traits and performance accuracy, which were most prominent when social information was volatile. Furthermore, it is consistent with the extensive amount of evidence indicating impairments in associative and social learning in psychopathy (Blair, 2007; Blair et al., 2004; Brazil et al., 2013a, b; Mitchell et al., 2006; Von Borries et al., 2010). Other model-based findings showed that the perceived trustworthiness of the advice was equally stable across information sources (social vs. nonsocial) and was unrelated to psychopathic traits. The relationship between perceived trustworthiness and psychopathic traits has yielded mixed findings in the literature (Gong et al., 2019; Richell et al., 2005; Mahaffey & Marcus, 2006). A post-hoc analysis of the present data showed no significant link between psychopathic traits and advice ratings (all *p* > 0.05; see also Fig. S4 in the Supplemental material), suggesting that advice taking was independent of the phase of the task. Together, these findings underscore the need for further research to clarify the complexities of this relationship and the conditions under which such associations might emerge.

The model parameter reflecting social bias showed that participants relied preferentially on social over nonsocial information, which was consistent with previous findings (Diaconescu et al., 2020). One striking finding is that we did not detect evidence for a relationship between the level of psychopathic traits and the preference for one of the information sources over the other when making choices (hypothesis 1). In other words, the general bias towards using social information was not associated with the level of psychopathic traits. This is in contrast with previous findings indicating that individuals with high levels of interpersonal-affective traits showed diminished use of social information during learning (Blair, 2013; Brazil et al., 2011, 2013a), while individuals with high levels of impulsive-antisocial traits, characterized with a hypersensitivity to reward, showed increased use of reward-based information (Buckholtz et al., 2010). Taken together, our behavioral results support the hypothesis that more pronounced levels of psychopathic traits co-occur with greater impairments in associative learning using social information and suggest that antisocial traits are linked to a reduced ability to adapt to changes in the reliability of social information. There was no evidence supporting that these impairments were related to a change in preference for one of the information sources or affected the confidence and risk that was taken to obtain a higher reward.

We performed planned comparisons and chose not to apply a correction for multiple comparisons to avoid overly conservative results. Instead, we conducted Bayesian analyses as a complementary check. It is important to note that the correlation between antisocial traits and performance accuracy in volatile trials was not significant based on the Bayesian confidence intervals. Therefore, we emphasize that these findings should be interpreted as exploratory. Future research is needed to replicate and validate these results.

Another important consideration is that, like many studies examining learning from multiple sources (e.g., Behrens et al., 2008), the current study used a task design in which learning from one source of information was directly tied to feedback (i.e., the correct card color), while learning from the other source was not. Specifically, in our study, learning from nonsocial information was directly linked to the accuracy of the card color, whereas the validity of the advice had to be inferred indirectly through the accuracy of the card color. Rybicki and colleagues (2022) recently emphasized the importance of distinguishing between primary learning (i.e., learning from one's own experience) and secondary learning (i.e., learning from an additional source), showing that dopaminergic mechanisms underlying learning can be differentiated along a primary-secondary axis rather than a social-individual one. In light of this work, our findings on psychopathic traits could be interpreted as diminished learning from secondary information rather than specifically from social information. Such an interpretation aligns more closely with previous research showing that psychopathic traits are associated with disruptions in both social and nonsocial learning (Brazil et al., 2013a, b). This underscores the need for future studies to use well-controlled task designs that can more precisely disentangle these different learning processes.

The increased precision offered by the modelling approach was enhanced further by examining electrophysiological activity in the theta band over the mPFC to obtain a direct measure of adaptive control during associative learning. Our results did not show any differences in midfrontal theta power during volatile compared with stable trials. This contrasts with our other finding, suggesting that the parameter that represents the rate of change in volatility (i.e., meta-volatility) for social information was positively related to theta activity. The latter finding provides evidence supporting the involvement of theta activity in the implementation of adaptive control during associative learning, as proposed previously (Behrens et al., 2007). Future studies including community samples are needed to examine this effect in more detail. Finally, we showed that, overall, theta activation was negatively related to psychopathic traits (total and facet scores). This corroborates our findings indicating a negative relationship between performance accuracy and psychopathic traits, and a negative relationship between theta power and performance accuracy. Meta-analytical findings showed that theta plays a key role in the processing of emotion and increased theta synchronization was found in individuals scoring high on measures of emotional intelligence (Knyazev et al., 2013). The lower theta power that we found in relation to psychopathic traits are consistent with the emotional deficits and decreased emotional intelligence that were found in individuals with high levels of psychopathic traits (Megías et al., 2018).

One limitation of the current study pertains to the sample type. We used the oversampling procedure to enhance the presence of extreme scores on both sides of the distribution of our community sample. Nevertheless, all participants that were included in this study were considered healthy and generally functioning well in society. It is yet unclear how findings from a community sample translate to clinical or forensic populations. With this in mind, we want to highlight the need to be careful in the generation of claims about “impairments’ based on our results and encourage replications of this experiment with clinical and offender samples.

A second limitation relates to the task characteristics. In this study, all participants completed the same trial order, with the advice remaining stable and valid for the first 49 trials. This may have contributed to the general preference for social information observed in our findings. One could argue that the associations between behavioral outcomes and psychopathic traits reflect a reduced ability or motivation to update beliefs about volatility after a period of stability, rather than specific difficulties in updating beliefs about social volatility. However, given that there is no indication of reduced learning ability in our sample (e.g., no relationship with education level) and that individuals with psychopathic traits, including offenders, exhibit typical basic reward learning (e.g., points), we consider this explanation unlikely.

To summarize, the present study identified potential sources of disturbances in associative learning as levels of psychopathic traits increase. In addition, it emphasizes the role of theta activation in these learning processes. In general, our results show that the increasing presence of psychopathic traits is 1) not associated with a preference for social or nonsocial information in our sample, 2) associated with diminished belief updating about other’s trustworthiness, and 3) associated with decreased theta activity in the mPFC during associative learning. An increased prevalence of antisocial traits, in particular, seems to be linked to a reduced ability to adapt to changes in the trustworthiness of social information. Decreased brain activity in the theta band was found to be linked to higher levels of psychopathic traits, and we observed indications that theta is involved in tracking the volatility of social information. While prior studies focused on the link between theta activity with either cognitive performance or psychopathic traits, we propose that there is a relationship between the three concepts. Finally, the results demonstrated that the impairments that were associated to increased levels of psychopathic traits, mostly present in the social domain, did not relate to a preference for one of the information sources, and did not affect the risk that was taken to obtain a high reward. The findings contribute to our understanding of the underlying mechanisms of social and nonsocial learning in psychopathy and might have implications for improving current treatment interventions, as the success of treatment partly relies on the patient's ability to incorporate and use information from previous experiences and information provided by therapists and their social environment.

## Supplementary Information

Below is the link to the electronic supplementary material.

Supplementary file1 (DOCX 7483 KB)

## Data Availability

The URL provided in the cover letter should only be shared with reviewers. The data will be publicly available upon manuscript acceptance.

## Code availability

Data are available on the Radboud Data Repository: 10.34973/db8vs911.

## Declarations

### Preregistration

The study was not preregistered.
