# Supplementary material for: Dissecting how psychopathic traits are linked to learning in different contexts: A multilevel computational and electrophysiological approach
Source: Cogn Affect Behav Neurosci. 2025 Jul 23;25(5):1543–62. doi: 10.3758/s13415-025-01295-z (PMC12464111; doi:10.3758/s13415-025-01295-z)
Supplement: Supplementary file 1 — Supplementary file1 (DOCX 7483 KB) [file 13415_2025_1295_MOESM1_ESM.docx]

**Supplemental material**

**Table S1.** Bayesian pairwise correlation between SRP scores and performance accuracy

|  | SRP total score | | SRP interpersonal | | SRP affective | | SRP lifestyle | | SRP Antisocial | |
| --- | --- | --- | --- | --- | --- | --- | --- | --- | --- | --- |
|  | *Mode r* | *CI* | *Mode r* | *CI* | *Mode r* | *CI* | *Mode r* | *CI* | *Mode r* | *CI* |
| Performance accuracy |  |  |  |  |  |  |  |  |  |  |
| Overall | -.198 | [-.39, .01] | -.146 | [-.34, .06] | -.146 | [-.34, .06] | -.246 | [-.43, -.04] | -.085 | [-.29, .13] |
| Stable | -.143 | [-.34, .07] | -.089 | [.29, .12] | -.097 | [-.30, .11] | -.222 | [-.41, -.02] | -.029 | [-.23, .18] |
| Volatile | -.235 | [-.43, -.04] | -.207 | [-.40, -.00] | -.185 | [-.38, .02] | -.249 | [-.43, -.04] | -.134 | [-.33, .08] |
| Non-social information |  |  |  |  |  |  |  |  |  |  |
| Stable | -.164 | [-.36, .04] | -.113 | [-.31, .10] | -.164 | [-.36, .05] | -.164 | [-.36, .05] | -.086 | [-.29, .12] |
| Volatile | -.115 | [-.32, .09] | -.088 | [-.29, .12] | -.043 | [-.25, .02] | -.186 | [-.38, .02] | -.037 | [-.24, .17] |
| Social information |  |  |  |  |  |  |  |  |  |  |
| Stable | -.064 | [-.27, .14] | -.031 | [-.24, .17] | -.001 | [-.21, .21] | -.173 | [-.37, .04] | .030 | [-.18, .24] |
| Volatile | -0.23 | [-.42, -.03] | -.209 | [-.40, -.00] | -.227 | [-.42, -.02] | -.157 | [-.35, .05] | -.158 | [-.35, .05] |
| *Note.* Asterisks indicate significant correlations (*p*<.05). | | | | | | | | | |  |

**Table S2.** Bayesian correlations between performance accuracy and computational parameters and mean theta power over the midfrontal and individual electrodes

|  | midfrontal | | Fz | | FCz | | Cz | |
| --- | --- | --- | --- | --- | --- | --- | --- | --- |
|  | *Mode r* | *CI* | *Mode r* | *CI* | *Mode r* | *CI* | *Mode r* | *CI* |
| Performance accuracy |  |  |  |  |  |  |  |  |
| Overall | .217 | [.00, .41] | .213 | [.00, .41] | .271 | [.06, .46] | .207 | [-.01, .40] |
| Stable | .127 | [-.09, .33] | .142 | [.07, .34] | .188 | [-.03, .39] | .110 | [-.11, .32] |
| Volatile | .314 | [.10, .50] | .277 | [.07, .46] | .343 | [.14, .52] | .309 | [.10, .49] |
| Non-social information |  |  |  |  |  |  |  |  |
| Kappa$(\kappa_{c}$) | -.085 | [-.30, .13] | -.120 | [-32, .10] | -.018 | [-23, .20] | -.108 | [-.31, .11] |
| Theta ($\vartheta_{c}$) | .056 | [-.16, .27] | <.001 | [-.21, .21] | .101 | [-.11, .31] | .065 | [-.15, .27] |
| Social information |  |  |  |  |  |  |  |  |
| Kappa $(\kappa_{a}$) | .203 | [-.01, .40] | .128 | [-.09, .33] | .215 | [.00, .41] | .203 | [-.01, .40] |
| Theta ($\vartheta_{a}$) | .224 | [.01, .42] | .154 | [-.06, .35] | .210 | [-.00, .40] | .214 | [.00, .41] |
| Note. Asterisks indicate significant correlations (***p*<.05). | | | |  |  |  |  |  |

**Table S3.** Bayesian correlations between SRP scores and mean theta power over the midfrontal and individual electrodes

|  | midfrontal | | Fz | | FCz | | Cz | |
| --- | --- | --- | --- | --- | --- | --- | --- | --- |
|  | *Mode r* | *CI* | *Mode r* | *CI* | *Mode r* | *CI* | *Mode r* | *CI* |
| SRP Interpersonal | -.325 | [-.51, -.12] | -.279 | [-.47, -.07] | -.340 | [-.52, -.13] | -.338 | [-.52, -.14] |
| SRP Affective | -.322 | [-.50, -.11] | -.247 | [-.44, -.03] | -.339 | [-.52, -.13] | -.375 | [-.57, -.17] |
| SRP Lifestyle | -.237 | [-.43, -.02] | -.179 | [-.38, -.04] | -.251 | [-.44, -.04] | -.277 | [-.46, -.06] |
| SRP Antisocial | -.180 | [.-.38, .04] | -.114 | [-.32, .10] | -.193 | [-.39, .02] | -.239 | [-.43, -.03] |
| SRP Total | -.332 | [-.51, -.13] | -.261 | [-.45, -.05] | -.350 | [-.53, -.14] | -.378 | [-.55, .18] |
| Note. Asterisks indicate significant correlations (***p*<.05). | | | |  |  |  |  |  |

|  | SRP total score | | SRP interpersonal | | SRP affective | | SRP lifestyle | | SRP Antisocial | |
| --- | --- | --- | --- | --- | --- | --- | --- | --- | --- | --- |
|  | *rho* | *95% CI* | *rho* | *95% CI* | *rho* | *95% CI* | *rho* | *95% CI* | *rho* | *95% CI* |
| Advice taking |  |  |  |  |  |  |  |  |  |  |
| Overall | .019 | [-.20, .23] | .101 | [-.12, .32] | .019 | [-.20, .22] | -.031 | [-.24, -.18] | .103 | [-.20, .23] |
| Stable | .017 | [-.22, .25] | .104 | [-.14, .32] | -.003 | [-.23, .21] | -.033 | [-.25, .19] | .064 | [-.18, .29] |
| Volatile | .012 | [-.22, .25] | .067 | [-.16, .30] | .023 | [-.20, .23] | -.016 | [-.24, .20] | .143 | [-.08, .35] |
| Non-social information |  |  |  |  |  |  |  |  |  |  |
| Stable | .047 | [-.21, .27] | .081 | [-.17, .26] | .054 | [-.21, .26] | .033 | [-.18, .31] | .078 | [-.21, .27] |
| Volatile | .081 | [-.13, .29] | .152 | [-.05, .34] | .056 | [-.15, .26] | .007 | [-.20, .24] | .156 | [-.07, .36] |
| Social information |  |  |  |  |  |  |  |  |  |  |
| Stable | .032 | [-.18, .24] | .138 | [-.07, .34] | -.005 | [-.21, .20] | -.040 | [-.25, .17] | .089 | [-.11, .30] |
| Volatile | .002 | [-.23, .21] | .017 | [-.22, .24] | .061 | [-.16, .26] | -.001 | [-.23, .21] | .085 | [-.16, .32] |
| *Note.* CI = confidence interval (1000 bootstrap samples). | | | | | | | | | |  |

**Table S4.** Spearman correlations between SRP scores and advice taking

**Table S5.** Overview of the parameters and their interpretation

| Parameter | Interpretation |
| --- | --- |
| $\kappa_{a}$ | coupling (i.e. information flow) between the second and third hierarchy levels of social information |
| $\kappa_{c}$ | coupling (i.e. information flow) between the second and third hierarchy levels of non-social information |
| $\vartheta_{a}$ | variance of the volatility (i.e., meta-volatility) of social information |
| $\vartheta_{c}$ | variance of the volatility (i.e., meta-volatility) of non-social information |
| $\zeta$ | the participant’s bias towards social information |

**Figure S3.** Comparison of 3-level HGF, 2-level HGF and Rescorla-Wagner models

**
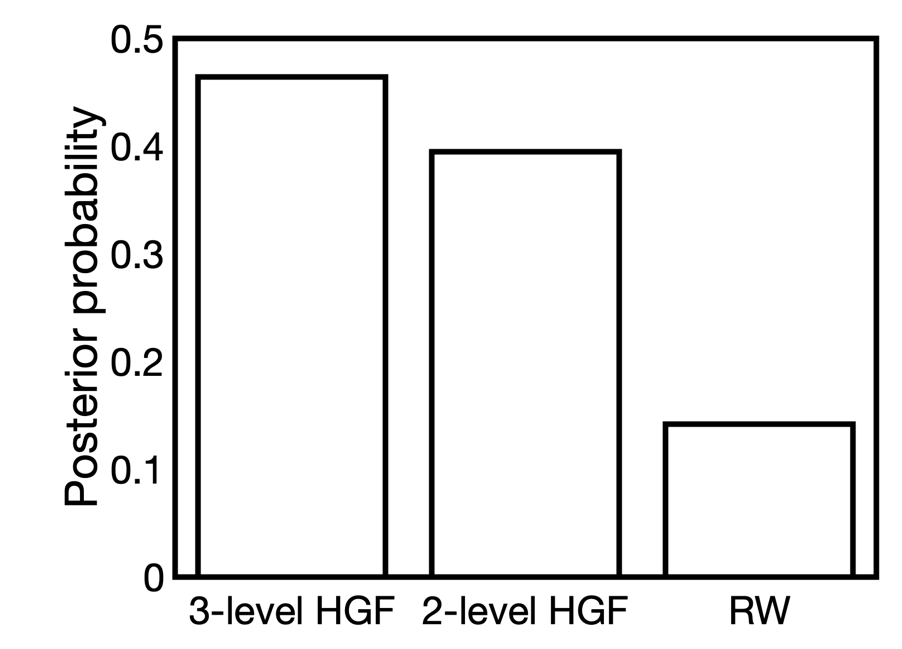
**

**Table S6.** Model comparison: Individual fit measures (per participant N=86)

| 3L_HGF | | 2L_HGF | | RW | |
| --- | --- | --- | --- | --- | --- |
| *BIC* | *LME* | *BIC* | *LME* | *BIC* | *LME* |
| 943.67 | -459.79 | 934.4 | -459.17 | 937.11 | -459.17 |
| 834.11 | -407.73 | 825.17 | -406.63 | 22397.65 | -406.63 |
| 828.13 | -417.09 | 861.05 | -432.57 | 872.36 | -432.57 |
| 871.73 | -428.28 | 880.81 | -435.18 | 872.77 | -435.18 |
| 986.91 | -481.46 | 979.56 | -479.97 | 969.95 | -479.97 |
| 1139.8 | -555.45 | 1129.8 | -554.89 | 1130.1 | -554.89 |
| 932.87 | -455.01 | 921.03 | -453.04 | 18600.5 | -453.04 |
| 875.08 | -426.49 | 868.19 | -426.88 | 878.9 | -426.88 |
| 894.56 | -435.04 | 886.81 | -435.64 | 884.64 | -435.64 |
| 857.31 | -418.96 | 848 | -418.05 | 21122.32 | -418.05 |
| 874.85 | -433.15 | 897.34 | -446.8 | 898.91 | -446.8 |
| 915.66 | -448.98 | 918.89 | -451.9 | 902.77 | -451.9 |
| 923.37 | -451.57 | 912.35 | -450.06 | 937.98 | -450.06 |
| 733.67 | -359.63 | 728.92 | -362.35 | 744.09 | -362.35 |
| 835.76 | -415.74 | 869.59 | -431.95 | 861.93 | -431.95 |
| 602.65 | -297.42 | 586.95 | -292.34 | 23299.49 | -292.34 |
| 1056.3 | -517.72 | 1058.48 | -520.62 | 1023.24 | -520.62 |
| 931 | -453.27 | 919.13 | -452.07 | 912.48 | -452.07 |
| 889.96 | -434.88 | 885.88 | -436.05 | 879.54 | -436.05 |
| 849.05 | -416.71 | 844.15 | -417.66 | 17359.35 | -417.66 |
| 949.76 | -462.88 | 942.05 | -462.58 | 936.21 | -462.58 |
| 858.87 | -420.4 | 857.72 | -423.22 | 868.76 | -423.22 |
| 953.27 | -466.95 | 943.95 | -465.42 | 17663.67 | -465.42 |
| 1082.05 | -528.04 | 1070.65 | -526.27 | 19843.64 | -526.27 |
| 776.99 | -383.35 | 809.85 | -399.33 | 796.03 | -399.33 |
| 1017.82 | -495.11 | 1014.87 | -496.98 | 1009.17 | -496.98 |
| 1049.03 | -512.15 | 1045.91 | -513.42 | 17716.65 | -513.42 |
| 941.12 | -461.89 | 939.84 | -463.44 | 953.05 | -463.44 |
| 1036.98 | -508.86 | 1035.77 | -511.03 | 1043.71 | -511.03 |
| 871.35 | -429.3 | 864.98 | -427.58 | 20914.42 | -427.58 |
| 1063.76 | -519.29 | 1052.7 | -517.96 | 20535.78 | -517.96 |
| 781.7 | -386.36 | 799.36 | -398.05 | 825.35 | -398.05 |
| 1139.73 | -557.72 | 1135.01 | -558.84 | 20181.83 | -558.84 |
| 961.67 | -469.48 | 956.89 | -470.34 | 942.43 | -470.34 |
| 929.6 | -454.01 | 922.4 | -453.97 | 20169.04 | -453.97 |
| 993.46 | -485.79 | 1022.34 | -500.48 | 939.87 | -500.48 |
| 928.2 | -452.82 | 922.73 | -452.6 | 915.37 | -452.6 |
| 743.72 | -364.25 | 737.97 | -365.47 | 750.13 | -365.47 |
| 1035.4 | -508.63 | 1034.25 | -510.77 | 1041.32 | -510.77 |
| 934.31 | -458.34 | 924.11 | -456.52 | 26445.72 | -456.52 |
| 834.08 | -410.09 | 859.23 | -422.77 | 817.75 | -422.77 |
| 736.05 | -358.9 | 742.26 | -364.82 | 749.24 | -364.82 |
| 944.65 | -462.85 | 948.52 | -465.63 | 904.82 | -465.63 |
| 604.18 | -293.98 | 597.18 | -294.27 | 599.39 | -294.27 |
| 1117.8 | -546.82 | 1108.28 | -545.9 | 18658.28 | -545.9 |
| 931.98 | -455.5 | 927.18 | -457.58 | 932.04 | -457.58 |
| 1117.91 | -548.7 | 1114.02 | -550.37 | 18819.23 | -550.37 |
| 984.83 | -481.38 | 981.06 | -481.67 | 982.11 | -481.67 |
| 1001.23 | -491.68 | 990.46 | -489.89 | 26840 | -489.89 |
| 1106.9 | -544.48 | 1093.09 | -541.3 | 22894.93 | -541.3 |
| 864.99 | -423.41 | 858.54 | -422.93 | 18449.51 | -422.93 |
| 954.38 | -468.17 | 958.87 | -471.2 | 939.24 | -471.2 |
| 1038.57 | -507.67 | 1034.15 | -508.34 | 1030.82 | -508.34 |
| 956.67 | -469.66 | 963.44 | -474.31 | 903.99 | -474.31 |
| 759.18 | -370.39 | 746.2 | -368.73 | 763.79 | -368.73 |
| 1009.79 | -498.81 | 1017.9 | -505.29 | 1021.2 | -505.29 |
| 984.35 | -481.27 | 980.99 | -482.74 | 962.95 | -482.74 |
| 990.17 | -483.14 | 975.61 | -478.75 | 22859.81 | -478.75 |
| 874.52 | -423.07 | 867.2 | -423.37 | 864.74 | -423.37 |
| 1030.14 | -509.33 | 1045.52 | -517 | 1027.3 | -517 |
| 887.9 | -437.31 | 889.95 | -439.5 | 17476.31 | -439.5 |
| 1009.04 | -493.55 | 1006.04 | -494.8 | 988.01 | -494.8 |
| 868.44 | -423.15 | 860.7 | -423.32 | 859.29 | -423.32 |
| 1044.36 | -511.93 | 1036.84 | -512.1 | 18454.48 | -512.1 |
| 891.14 | -441.05 | 900.16 | -447.43 | 17049.47 | -447.43 |
| 853.35 | -417.6 | 842.6 | -415.68 | 24376.3 | -415.68 |
| 725.22 | -353.46 | 710.99 | -350.15 | 20590.24 | -350.15 |
| 995.65 | -503.83 | 962.98 | -473.54 | 22284.84 | -473.54 |
| 863.57 | -424.71 | 853.87 | -423.07 | 21893.16 | -423.07 |
| 806.71 | -392.88 | 792.27 | -388.1 | 778.65 | -388.1 |
| 787.26 | -388.04 | 778.57 | -386.65 | 22986.4 | -386.65 |
| 832.9 | -409.57 | 836.49 | -411.63 | 841.47 | -411.63 |
| 989.53 | -483.86 | 977.04 | -481.62 | 22417.33 | -481.62 |
| 1009.73 | -492.1 | 1004.76 | -493.02 | 993.45 | -493.02 |
| 1020.52 | -497.79 | 1010.64 | -497.36 | 19923.5 | -497.36 |
| 885.71 | -433.31 | 890.55 | -437.33 | 883.73 | -437.33 |
| 875.57 | -430.31 | 893.39 | -440.3 | 890.97 | -440.3 |
| 844.1 | -412.33 | 833.6 | -411.22 | 840.48 | -411.22 |
| 1011.85 | -495.64 | 1000.11 | -493.2 | 19650.3 | -493.2 |
| 745.69 | -365.11 | 732.81 | -363.45 | 743.01 | -363.45 |
| 625.62 | -309.88 | 651.32 | -322.69 | 597.59 | -322.69 |
| 819.75 | -401.15 | 808.77 | -399.32 | 21439.11 | -399.32 |
| 1000.85 | -489.75 | 991.56 | -488.32 | 20763.64 | -488.32 |
| 952.26 | -466.88 | 939.84 | -464.13 | 21147.19 | -464.13 |
| 850.87 | -416.44 | 839.9 | -415.08 | 24284.94 | -415.08 |
| 1005.96 | -493.52 | 1000.1 | -493.88 | 1004.71 | -493.88 |

*Note.* 2L-HGF= 2-level Hierarchical Gaussian Filter model, 3L-HGF = 3-level Hierarchical Gaussian Filter model, RW = Rescorla-Wagner model

**Figure S1.** Parameter recovery check: Simulated (black) and the true (blue) choice trajectory **
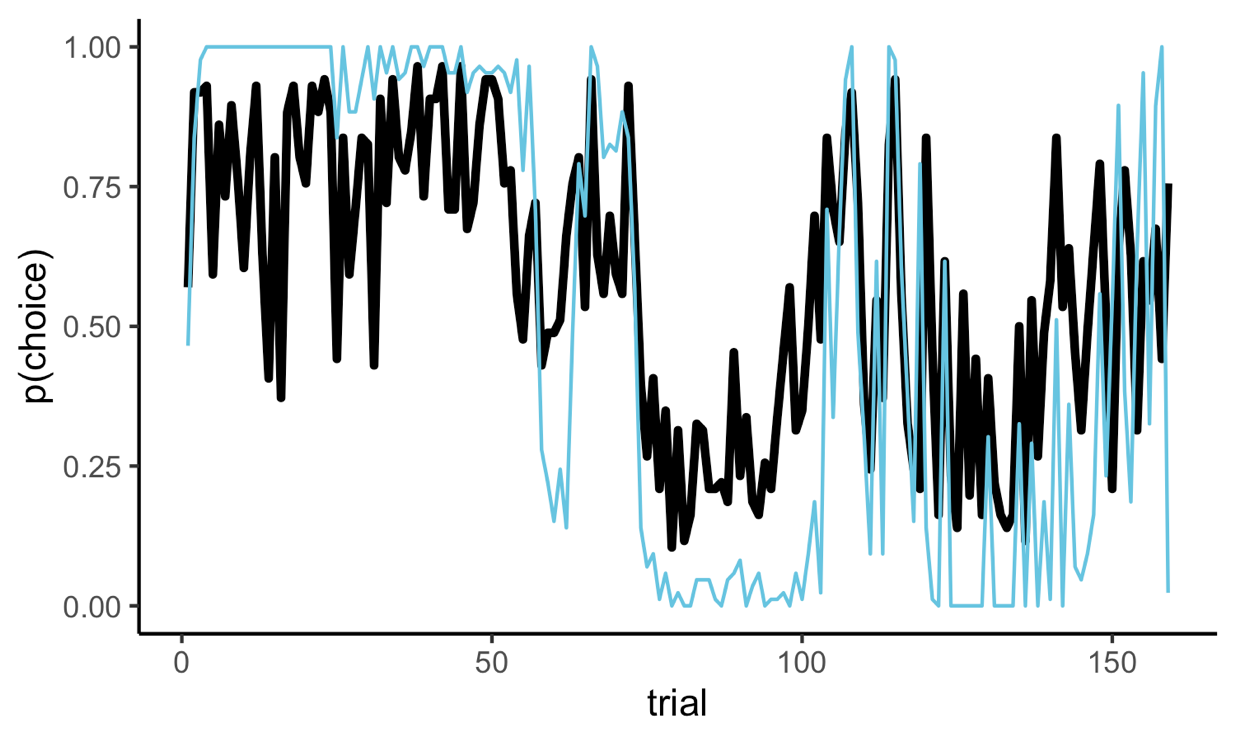
**

**Figure S2.** Parameter recovery check: Simulated (black) and the true (blue) wager trajectory
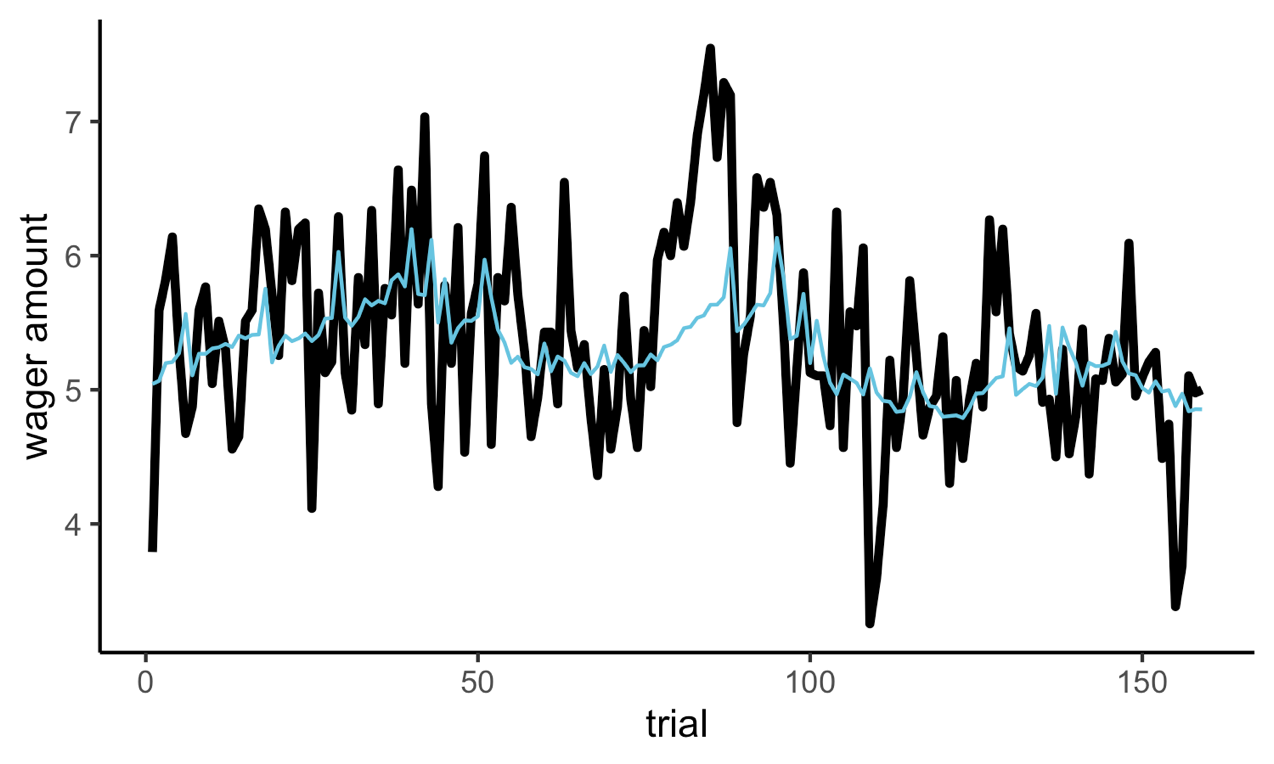


**Table S7. Bayesian zero-order correlations: Parameter recovery**

| Behaviour | Correlation | 95% CI |
| --- | --- | --- |
| Win-stay | .45 | [.27, .60] |
| Lose-shift | .58 | [.44, .72] |
| Mean wager | .99 | [.98, .99] |

**Table S8. Bayesian zero-order correlations: Parameter recovery**

| Parameter | Correlation | 95% CI |
| --- | --- | --- |
| Ka_r | .37 | [.18, .53] |
| Ka_a | .73 | [.62, .82] |
| Th_r | .28 | [.08, .45] |
| Th_a | .48 | [.32, .63] |
| zeta | .93 | [.88, .95] |
